# Overlapping Myocarditis and Postural Orthostatic Tachycardia Syndrome After COVID-19 Messenger RNA Vaccination: A Case Report

**DOI:** 10.7759/cureus.31006

**Published:** 2022-11-02

**Authors:** Yuichi Sanada, Junji Azuma, Yasuhiro Hirano, Yasuhiro Hasegawa, Takehisa Yamamoto

**Affiliations:** 1 Pediatrics, Minoh City Hospital, Osaka, JPN

**Keywords:** droxidopa, postural orthostatic tachycardia syndrome, myocarditis, vaccine, covid-19

## Abstract

The worldwide spread of the coronavirus disease 2019 (COVID-19) pandemic and the significant morbidity and mortality rate associated with it led to the rapid development of several COVID-19 vaccines. While serious side effects related to the vaccines are rare, various adverse events have been reported to occur after COVID-19 messenger RNA (mRNA) vaccination, including myocarditis, Guillain-Barré syndrome, and thrombosis. Postural orthostatic tachycardia syndrome (POTS) is a chronic cardiovascular dysautonomia among young and middle-aged individuals. Although the pathophysiology of POTS is thought to be heterogeneous, vaccine-induced immune-mediated autonomic dysfunction is hypothesized to be one cause of the syndrome.

In this report, we present a case of myocarditis and POTS occurring in a 13-year-old male following COVID-19 mRNA vaccination. He presented with persistent severe fatigue and headache. The patient's symptoms improved after intravenous immunoglobulin for myocarditis, non-pharmacologic interventions, and multiple medications for POTS.

## Introduction

The coronavirus disease 2019 (COVID-19) pandemic, which has put an enormous burden on global healthcare systems and caused numerous fatalities worldwide, has led to the development of COVID-19 vaccines by various companies/organizations. The Pfizer-BioNTech vaccine is based on modified messenger RNA (mRNA) that encodes the severe acute respiratory syndrome coronavirus 2 (SARS-CoV-2) spike proteins on the outer surface of the virus. Of Pfizer-BioNTech COVID-19 vaccine recipients who are aged 12-15 years, 90.7% have experienced at least one systemic reaction within seven days of receiving the vaccination [1]. Fatigue, headache, chills, and new or worsened muscle pain have been common, but in most cases, symptoms are mild or moderate and resolve within a few days. Severe fatigue and headache after a second dose of the vaccine have been reported in only 2.4% and 2% of recipients respectively [1]. Serious side effects are rare, but various adverse events, including myocarditis, Guillain-Barré syndrome, and thrombosis, have been described [2].

Postural orthostatic tachycardia syndrome (POTS) is a chronic autonomic cardiovascular disorder observed in young and middle-aged individuals. It is characterized by chronic orthostatic intolerance, an abnormal increase in heart rate on standing, and physical deconditioning [3-6]. Although the pathophysiology of POTS is thought to be heterogeneous, vaccine-induced immune-mediated autonomic dysfunction is hypothesized to be one cause of the syndrome [3].

Here, we describe a pediatric patient with myocarditis and POTS who complained of persistent severe fatigue and headache on the day after receiving the second dose of COVID-19 mRNA vaccination.

## Case presentation

The patient was a previously healthy 13-year-old male who developed fever, headache, fatigue, and sleep disturbance on the day after receiving a second dose of the Pfizer-BioNTech COVID-19 mRNA vaccine. He presented to our hospital on the 14th day after the symptoms began. His vital signs, heart sounds, and neurologic findings were normal. Acetaminophen, ibuprofen, and Chinese herbs (Goreisan and Kakkonto) were started for the headache and fatigue. However, the patient’s symptoms persisted, and he felt unable to get out of bed all day. An active standing test (AST) showed that when the patient stood, his heart rate immediately increased from 65 to 140 beats/minute (bpm). Based on the current diagnostic criteria [3-6], we diagnosed POTS. The patient was educated about non-pharmacologic treatments such as the need to increase salt and fluid intake and exercise; he was also started on midodrine (2 mg, twice daily). However, on the 33rd day after vaccination, he was admitted to our hospital due to severe fatigue, headache, and orthostatic symptoms including lightheadedness and palpitations.

**Figure 1:**
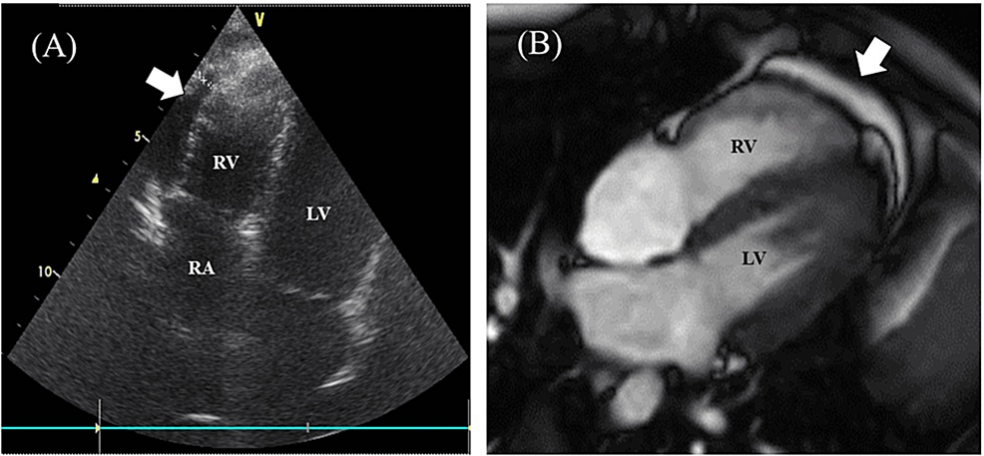
Echocardiogram and cardiac four-chamber cine magnetic resonance image (A) Echocardiogram obtained on the 33rd day after a second COVID-19 messenger RNA vaccination. Pericardial effusion is observed at the right ventricular side. (B) Cardiac four-chamber cine magnetic resonance image obtained on the 46th day after vaccination. Pericardial effusion is observed, but no abnormalities in the myocardium were found RA: right atrium; RV: right ventricle; LV: left ventricle

The blood tests revealed slight increases in creatine kinase-MB (11 ng/mL; normal range: 0-6 ng/mL) and troponin I (33.8 pg/mL; normal range: 0-30 pg/mL). Thyroid-stimulating hormone, free T3, and free T4 were within normal range. Antinuclear antibodies and antineutrophil cytoplasmic antibodies were negative. Electrocardiography showed no abnormalities, but echocardiography revealed a slight pericardial effusion (3-5 mm) at the right anterior ventricle, with a 72% normal ejection fraction (Figure 1A). Cardiac MRI showed a slight pericardial effusion without myocardial inflammation (Figure 1B). Brain MRI was normal. Those findings led to a diagnosis of mild myocarditis.

The patient was advised to take rest for the myocarditis, and 1.5 L intravenous saline daily was administered for the POTS. In addition, pregabalin (100 mg, twice daily) was started for the headache, and ramelteon was initiated for sleep disturbance. Pregabalin reduced the headache slightly, but the other symptoms remained. On the 75th day after vaccination, intravenous immunoglobulin (IVIG) 2 g/kg was administered for the myocarditis. A few days later, the patient’s symptoms partly improved, and he was able to walk for several kilometers, but only at night. Creatine kinase-MB and troponin I then normalized, and pericardial fluid was no longer detectable on echocardiography. However, severe fatigue and headache from morning to evening persisted.

**Figure 2:**
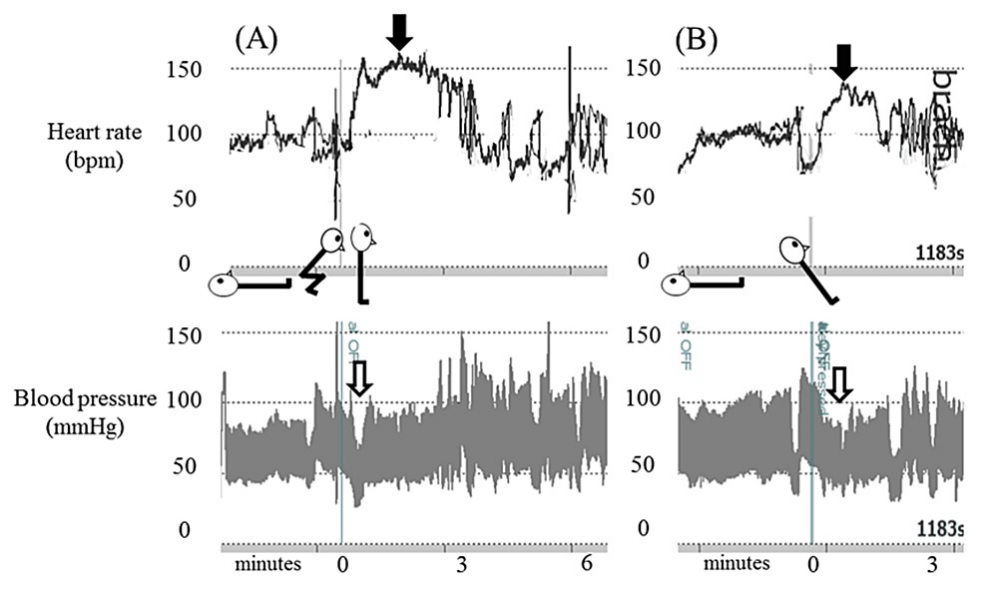
Active standing test and head-up tilt test (A) Active standing test. (B) Head-up tilt test. Black arrows indicate the marked increase in heart rate during orthostasis. White arrows indicate a transient decrease in blood pressure immediately after the patient stood up

On the 210th day after vaccination, the patient was referred to a tertiary center. Acetylcholine antibody and anti-ganglioside GM1 and anti-GQ1b antibody test results were normal. Motor nerve and sensory nerve conduction studies showed no abnormalities. AST (Figure 2A) and head-up tilt test (Figure 2B) demonstrated an increase in heart rate in excess of 40 bpm. Although the patient’s systolic and diastolic blood pressure (BP) immediately decreased from 95/54 mmHg to 70/40 mmHg when he stood up, that decrease did not persist and resolved within a few seconds (Figures 2A, 2B). Additionally, the decrease did not meet the criteria of initial orthostatic hypotension (a decrease in systolic BP exceeding 40 mmHg or a decrease in diastolic BP exceeding 20 mmHg) [5]. The diagnosis of POTS was confirmed.

The patient then received strict re-education in non-pharmacologic treatments. Chinese herbs and pregabalin were discontinued. The dose of midodrine was increased from 2 to 6 mg. Additionally, propranolol (10 mg, twice daily) on the 227th day and droxidopa (200 mg, twice daily) on the 234th day were added. Propranolol improved the orthostatic symptoms, and droxidopa improved the headache and fatigue, finally enabling the patient to be active at mid-day.

## Discussion

Several cases of myocarditis, pericarditis, or myopericarditis have been reported in individuals following mRNA vaccination for COVID-19 [7,8]. Because the clinical manifestations of heart inflammation have varied, applying the definitions proposed by the US Centers for Disease Control and Prevention for myocarditis, pericarditis, and myopericarditis is essential [7]. Based on these definitions, our case was compatible with myocarditis. Truong et al. have reported that the most common symptom of myocarditis associated with COVID-19 mRNA vaccination is chest pain (99.3%), with headache and fatigue occurring in 15.8% and 7.9% of cases respectively [8]. In most cases, the illness is mild and resolves clinically within a few days to a few weeks.

Optimal treatment strategies for COVID-19 vaccine-associated myocarditis are yet to be established. In previous reports, treatments have ranged from no anti-inflammatory therapies to glucocorticoids or IVIG [7,8]. In our case, IVIG administration led to partial improvement in the patient’s symptoms, normalization of serum creatine kinase-MB and troponin I, and dispersion of pericardial fluid. Nevertheless, the patient’s severe fatigue and headache persisted. Although various disorders can lead to POTS or POTS-like symptoms [9], no reports have yet suggested that myocarditis can present with POTS-like symptoms. In fact, making a definitive determination about whether myocarditis induced POTS-like symptoms or whether vaccination induced both myocarditis and POTS in our case is difficult. However, we speculate that our patient’s persistent symptoms derived mainly from POTS, because his myocarditis improved after IVIG treatment, and the remaining symptoms were typical of POTS. Additionally, no other causes of sinus tachycardia or fatigue were identified, including anemia, dehydration, hyperthyroidism, collagen disease, vasculitis, myasthenia gravis, and Guillain-Barré syndrome.

POTS is a clinical syndrome of orthostatic intolerance lasting at least three months and is associated with excessive upright tachycardia in the absence of sustained orthostatic hypotension [5,6]. The current diagnostic criteria define excessive tachycardia as an increase in the heart rate of at least 40 bpm for individuals who are 12-19 years of age during the first 10 minutes after taking an upright position [6]. Other symptoms of POTS are lightheadedness, palpitations, tremulousness, weakness, blurred vision, exercise intolerance, and fatigue. However, patients with POTS can present with many other symptoms as well, including headache and sleep disturbance [3-6]. Additionally, many patients report that their symptoms are worse in the morning and improve during the course of the day, as in our case [10]. Little is known about the long-term prognosis for pediatric patients with POTS. A 2019 study of long-term outcomes from China demonstrated that 48.4% of pediatric patients with POTS were free of symptoms at the one-year follow-up, with 85.6% being symptom-free after six years [11].

Physiologic mechanisms underlying POTS are thought to be heterogeneous. However, POTS can be precipitated by immunologic stressors such as viral infection or vaccination [3]. Notably, reports of adult cases of new-onset POTS after COVID-19 mRNA vaccination have been emerging [12-15]. Although the exact mechanism of vaccine-induced POTS is not clear, the autoimmune pathophysiology might be a key component [13-15]. To the best of our knowledge, no pediatric POTS cases after COVID-19 mRNA vaccination have yet been reported. However, such POTS cases might increase as pediatric COVID-19 mRNA vaccination rates increase in the future.

Available management protocols for POTS include a combination of non-pharmacologic measures such as exercise and increased intake of fluids and salt, and the use of medications [3-6]. The patient in our case received midodrine, propranolol, and droxidopa as pharmacotherapy for POTS. The synthetic amino acid analog droxidopa is metabolized to norepinephrine. The only published clinical experience with droxidopa in POTS found that this agent did not significantly affect BP, heart rate, or quality of life, but did improve orthostatic symptoms [3,4]. In our case, a combination of midodrine, propranolol, and droxidopa in addition to non-pharmacologic interventions improved the patient’s orthostatic symptoms and quality of life.

## Conclusions

We reported a pediatric case of myocarditis and POTS after COVID-19 mRNA vaccination that required IVIG administration and a combination of non-pharmacologic measures and multiple medications to resolve. Although the major adverse events of the Pfizer-BioNTech COVID-19 mRNA vaccine include fatigue and headache, the duration of symptoms mostly lasts only a few days. Therefore, we emphasize the importance of performing an echocardiogram and AST or a head-up tilt test when recipients of the vaccine complain of chronic deconditioning. Further studies are needed to elucidate the associations of COVID-19 mRNA vaccination with myocarditis and POTS.
